# Artificial Cornea Substitute Based on Hydrogel Skeletons with Natural Stromal Hierarchical Structure and Extracellular Matrix for Sutureless Transplantation

**DOI:** 10.1002/advs.202411540

**Published:** 2025-01-24

**Authors:** Long Zhao, Zhen Shi, Xiaoyu Zhang, Jingting Wang, Shang Yang, Fuyan Wang, Tan Li, Qingjun Zhou, Ting Wang, Weiyun Shi

**Affiliations:** ^1^ State Key Laboratory Cultivation Base Shandong Provincial Key Laboratory of Ophthalmology Eye Institute of Shandong First Medical University Qingdao 266071 China; ^2^ Binzhou Medical University Binzhou 264003 China; ^3^ Eye Institute of Shandong First Medical University Eye Hospital of Shandong First Medical University (Shandong Eye Hospital) Jinan 250021 China

**Keywords:** artificial cornea substitutes, biostructure replication, corneal extracellular matrix, hydrogel skeletons, sutureless transplantations

## Abstract

Corneal substitutes with structural and compositional characteristics resembling those of natural corneas have attracted considerable attention. However, biomimicking the complex hierarchical organization of corneal stroma is challenging. In this study, humanized corneal stroma‐like adhesive patches (HCSPs) are prepared through a multi‐step process. First, polyethylene glycol diacrylate is cast and cured within decellularized porcine cornea (DPC) templates. The DPCs are then enzymatically digested to obtain hydrogel skeletons, which are finally integrated with human corneal extracellular matrix and methacrylate gelatin. HCSPs replicate the ultrastructure, protein components, and optical properties of human corneas and exhibit improved anti‐swelling and anti‐degradation capabilities compared with conventional DPCs and recombinant human collagen patches. HCSPs can deliver methacrylate gelatin at the ocular surface temperature (37 °C) and achieve stable adhesion to the corneal stroma upon 405 nm light irradiation. Furthermore, HCSPs promote the survival and migration of corneal epithelial and stromal cells while preserving their phenotypes. In rabbit models of lamellar keratoplasty and microperforation repair, HCSPs accelerate epithelial healing, minimize suture‐associated complications, and maintain structural stability. These findings suggest that HCSPs are promising donor corneal substitutes for clinical applications.

## Graphical abstract

Humanized corneal stroma‐like adhesive patches (HCSPs) are developed for lamellar keratoplasty and corneal perforation repair. HCSPs exhibit histological, optical, and biomechanical characteristics similar to those of the natural cornea, with improved wet tissue adhesion, anti‐degradation, and anti‐swelling capabilities compared to conventional tissue‐engineered corneas. Their application in vivo simplifies surgical processes, minimizes post‐operative complications, and ensures graft stability.

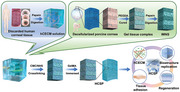

## 1 Introduction

**Figure 1:**
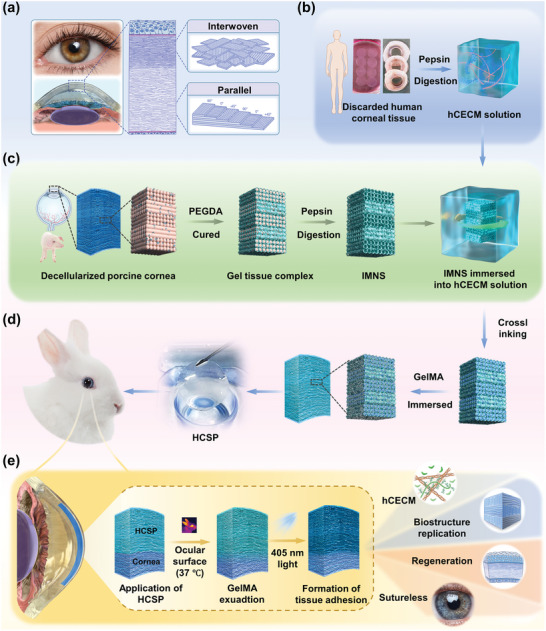
Preparation, adhesion mechanism, and biological characteristics of hCECM‐integrated corneal stroma‐like adhesive patch (HCSP). a) Illustration of the anatomical structure of corneal stroma. b) Schematic of human corneal extracellular matrix (hCECM) solution preparation. c) Schematic of inverted matrix fiber nanotubular hydrogel skeleton (IMNS) fabrication. d) Schematic of HCSP construction. e) Adhesion mechanism and applications of HCSP.

The corneal stroma is composed of 200–250 layers of regularly arranged lamellae, with the anterior stroma consisting of interwoven unidirectional fibril lamellae and the posterior stroma consisting of parallel fibril lamellae (**Figure** **1**a).^[^ ^1^ ^]^ This highly ordered structure of the corneal stroma is essential for maintaining optical clarity and mechanical strength. Damage to the stroma often results in corneal scarring and perforation and is a leading cause of blindness.^[^ ^2^ ^]^ Owing to the limited self‐regenerative potential of human corneas, donor corneal transplantation remains the gold standard treatment for corneal blindness.^[^ ^3^ ^]^ However, the sources of donor corneas are severely limited, with only one available for every 70 individuals among the ≈12.7 million population awaiting donor corneas worldwide.^[^ ^4^ ^]^

In recent years, various biomimetic corneal patches have been developed using advanced assembly techniques, such as 3D printing, electrospinning, self‐assembly, and electro‐assembly.^[^ ^5^ ^]^ Replicating the ordered structure of the corneal stroma has been proven to enhance optical performance and support the maintenance of keratocyte phenotypes.^[^ ^6^ ^]^ However, despite significant progress, replicating the complex ultrastructure and components of native corneal stroma remains a formidable challenge.

Considering the biological similarities between porcine and human corneas, decellularized porcine corneas (DPCs) have been introduced into clinical practice.^[^ ^7^ ^]^ However, immune rejection induced by xenotransplantation, including graft dissolution and calcification, remains a critical challenge.^[^ ^8^ ^]^ Additionally, the potential risk of infection due to cross‐species transmission of pathogens has raised concerns.^[^ ^9^ ^]^

Approximately 70% of the patients experience suture‐related complications after traditional corneal transplantation.^[^ ^10^ ^]^ In recent years, several in situ curable adhesives, including photocurable,^[^ ^11^ ^]^ bio‐orthogonally cross‐linked,^[^ ^12^ ^]^ ionically cross‐linked,^[^ ^13^ ^]^ and fibrin glue‐based adhesives,^[^ ^14^ ^]^ have been developed for sutureless repair. Photocurable adhesives, such as gelatin methacrylate (GelMA), are known for their efficiency and controllability, as they do not require multi‐component mixing and can be cured within seconds, although anesthesia may be required to alleviate discomfort from light exposure.^[^ ^11^, ^15^ ^]^ In addition, liquid adhesives can quickly seal corneal microperforations and minimize endothelial damage, compared to full‐thickness corneal transplantation.^[^ ^14^, ^16^ ^]^ However, the administration of adhesives presents a risk of iris synechiae due to their entry into the anterior chamber through perforation.^[^ ^11d^ ^]^

Despite the severe shortage of human donor corneas, tens of thousands of human corneal stromal fragments are discarded annually as surgical waste.^[^ ^17^ ^]^ In this study, healthy lenticules from refractive surgery were collected and digested to obtain a human corneal extracellular matrix (hCECM) solution (Figure 1b). Although the gelation of ECM solutions can be achieved by adding cross‐linkers or by spontaneous solidification, such hydrogels lack hierarchical structures and exhibit poor transparency.^[^ ^18^, ^19^ ^]^ To address these limitations, a novel strategy was designed to preserve the structural features of DPC while eliminating xenogeneic proteins by replacing them with hCECM, thereby realizing a tissue‐level “switch”. This approach provided hCECM with structural cues from the natural stroma and mitigated the risk of xenogeneic rejection.

First, polyethylene glycol diacrylate (PEGDA) was infiltrated into the interstices of DPC matrix fibers under vacuum, followed by photocuring under 365 nm irradiation. The gel‐tissue composite was then digested with pepsin to remove the DPCs while retaining the inverted matrix fiber nanotubular hydrogel skeleton (IMNS; Figure 1c; Figures S1a,b and S2 Supporting Information). Next, hCECM was integrated into the IMNS via vacuum‐assisted infiltration and chemical cross‐linking (Figure 1d; Figure S1c, Supporting Information). Finally, an hCECM‐integrated corneal stroma‐like adhesive patch (HCSP) was prepared by combining with GelMA (Figure 1d).

The 20% GelMA is thermosensitive, remains a free‐flowing liquid at 37 °C, and undergoes reversible gelation at 20 °C (Figure S3, Supporting Information). Owing to this property, 20% GelMA was infiltrated into the HCSP matrix at 37 °C and solidified by storage at < 20 °C (Figure 1d). When HCSPs were applied to the ocular surface (37 °C), GelMA re‐liquefied and exuded. Following 405 nm light exposure, GelMA rapidly polymerized into an interpenetrating polymer network between the recipient bed and HCSP, producing a tight bond (Figure 1e).

To evaluate the clinical potential of HCSPs, their ultrastructural, proteomic, and physical properties were characterized. Adhesive strength, shear strength, and burst pressure were quantified in vitro. The cytocompatibility of HCSPs and their influence on the regulation of corneal epithelial and stromal cell phenotypes were also assessed. Finally, the performance of the HCSPs in rabbit lamellar keratoplasty and microperforation repair was evaluated in vivo.

## 2 Results and Discussion

### 2.1 Structural and Compositional Characterization of HCSPs

**Figure 2:**
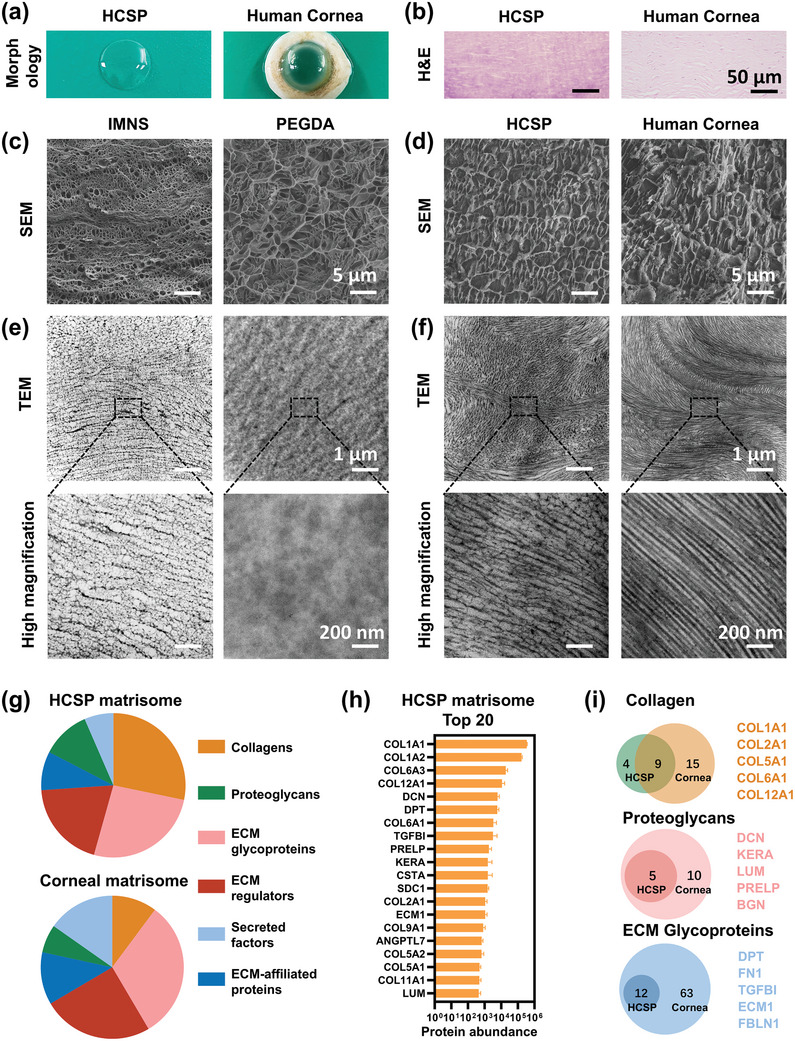
Morphology, architecture, and composition of HCSPs. a) Microscopic images showing morphology and macroscopic transparency of HCSPs and human cornea. b) Microscopic images of HCSPs and human corneal stroma obtained after H&E staining; scale bars = 50 µm. c) SEM images of IMNS and free polymerized PEGDA; scale bars = 5 µm. d) SEM images of HCSPs and human corneal stroma; scale bars = 5 µm. e) TEM images of IMNS and free‐polymerized PEGDA. f) TEM images of HCSPs and human corneal stroma. Scale bars of TEM images = 1 µm for low magnification and 200 nm for high magnification. g) Subtypes of matrisome proteins in HCSPs and human corneal stroma. h) Top 20 matrisome proteins in HCSPs. i) Number of core matrisome proteins detected in HCSPs and human corneal stroma.

The HCSPs exhibited good transparency and a natural curvature (**Figure** **2**a). Hematoxylin and eosin (H&E) staining indicated that the HCSPs had a microstructure similar to that of the human cornea (Figure 2b). Cryo‐scanning electron microscopy (cryo‐SEM) was performed to further assess the ultrastructure of HCSPs. Compared to the free polymerized PEGDA hydrogel, the porous structure of the IMNS exhibited a regular arrangement with alternating micropores of different sizes (Figure 2c). Upon immersion of the hCECM into the IMNS, the HCSPs exhibited corneal stroma‐like features, and their porous structures were filled (Figure 2d). These findings corroborate the nanoscale observations using transmission electron microscopy (TEM). The IMNS consisted of orthogonally arranged nanotubules with alternating cross and longitudinal sections (Figure 2e). In contrast, the free‐polymerized PEGDA had a homogeneous structure. When the hCECM was integrated into the IMNS, the HCSPs exhibited increased electron density and a lamellar‐like structure, similar to that of the natural stroma (Figure 2f).

Quantitative results showed that HCSPs were composed of ≈14.98 ± 0.33% PEGDA, 4.71 ± 0.38% hCECM, 3.58 ± 0.37% GelMA, and 76.73 ± 1.07% water. (Figure S4, Supporting Information). To compare the protein components of HCSPs with those of the human cornea, proteomic analyses were performed using the Human Protein Atlas. For a detailed characterization of hCECM, the matrisome database, an ensemble of genes encoding ECM and ECM‐associated proteins, was employed.^[^ ^20^ ^]^ The subtypes of matrisome proteins within HCSPs were similar to those in the human corneal stroma (Figure 2g). HCSPs contained abundant collagen, ECM glycoproteins, and proteoglycans (Figure 2h). The variety of matrisome proteins in hCECM was lower than that in the human cornea, which may be attributed to protein loss during decellularization, digestion, and perfusion (Figure 2i). Nevertheless, HCSPs shared most of the core matrisome components with the native cornea, including type I collagen, decorin, keratocan, lumican, and fibronectin (Figure 2i). These components play a crucial role in maintaining corneal transparency and promoting wound healing.^[^ ^21^ ^]^

### 2.2 Anti‐Swelling, Degradation Resistance, and Mechanical Properties of HCSPs

**Figure 3:**
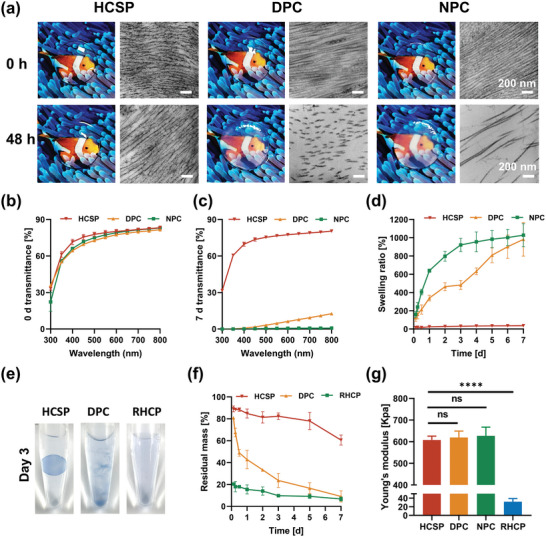
Physical properties of HCSP. a) Morphology and ultrastructure of HCSPs, DPCs, and NPCs after 0 and 48 h of immersion in artificial tears. (DPC: decellularized porcine cornea; NPC: natural porcine cornea); scale bars = 200 nm for TEM images. Light transmittance of HCSPs, DPCs, and NPCs at b) 0 d and c) 7 d of immersion in artificial tears. d) Swelling ratio. e) Physical states of HCSPs, DPCs, and RHCPs after incubation in collagenase solution for 3 d. For visualization, materials were stained with 0.4% trypan blue. (RHCP: recombinant human collagen patch) f) Percentage of residual mass. g) Results of mechanical strength test. Data represented as mean ± SD. ****, *p* < 0.0001. ns, not significant.

In healthy physiological states, the corneal stroma remains relatively dehydrated, as it is isolated from tears and aqueous humor by the epithelial and endothelial layers. However, in cases of corneal injury, particularly perforations, the absence of epithelial and endothelial layers directly exposes the graft to a fluid environment. Therefore, implants must possess anti‐swelling properties to maintain both their thickness and transparency. The swelling behavior of HCSPs in artificial tears was assessed and compared to that of DPCs and natural porcine corneas (NPCs). After 48 h of immersion, the HCSPs retained high transparency, with the IMNS exhibiting a tight and parallel alignment, resembling its pre‐immersion state (**Figure** **3**a). In contrast, DPCs and NPCs exhibited edema, reduced transparency, and stromal fiber breakage. Quantitative analysis revealed that after 7 d of immersion, the HCSPs maintained ≈80% transmittance in the visible spectrum (Figure 3b,c). During the testing period, HCSP exhibited relatively low swelling, with a swelling ratio of 36.9 ± 7.2% after 7 d. In comparison, the swelling ratios of DPCs and NPCs were 982.5 ± 183.9% and 1028.8 ± 125.8%, respectively (Figure 3d).

To evaluate the resistance of HCSPs to collagenase degradation, a high‐enzyme activity assay that surpassed physiological conditions was performed. DPCs and recombinant human collagen patches (RHCPs) in clinical trials, were used as controls.^[^ ^7^, ^22^ ^]^ After 3 d of incubation at 37 °C, the HCSPs maintained a cornea‐like morphology, whereas the DPCs underwent structural disintegration, and the RHCPs completely dissolved (Figure 3e). Residual mass analysis indicated that the HCSPs exhibited the lowest degradation rate during the incubation period, with a residual mass of 60.6 ± 4.6% after 7 d. In comparison, both the DPCs and RHCPs revealed final residual masses of less than 10% (Figure 3f). The PEGDA skeleton in HCSP may play a crucial role in resisting collagenase degradation and maintaining structural integrity (Figure S5, Supporting Information).

After transplantation, DPCs have shown early post‐operative edema and stromal thickness reduction during long‐term follow‐up in human trials.^[^ ^8^, ^23^ ^]^ Similarly, RHCPs failed to improve corneal thickness owing to their susceptibility to enzymatic degradation.^[^ ^24^ ^]^ The improved resistance to swelling and degradation demonstrated by the HCSPs is expected to provide more stable in vivo therapeutic effects.

The results of the tensile tests revealed that Young's modulus of the HCSPs reached 607.6 ± 18.5 KPa, comparable to that of the NPCs and DPCs (Figure 3g). The HCSPs exhibited a significantly higher Young's modulus than that of the RHCPs (32.4 ± 7.4 KPa; *P* = 9.93 × 10^−5^). Additionally, the tensile strength and toughness of the HCSPs were 16 and 24 times greater than those of RHCPs, respectively (Figure S6, Supporting Information).

### 2.3 Tissue Adhesion of HCSPs

**Figure 4:**
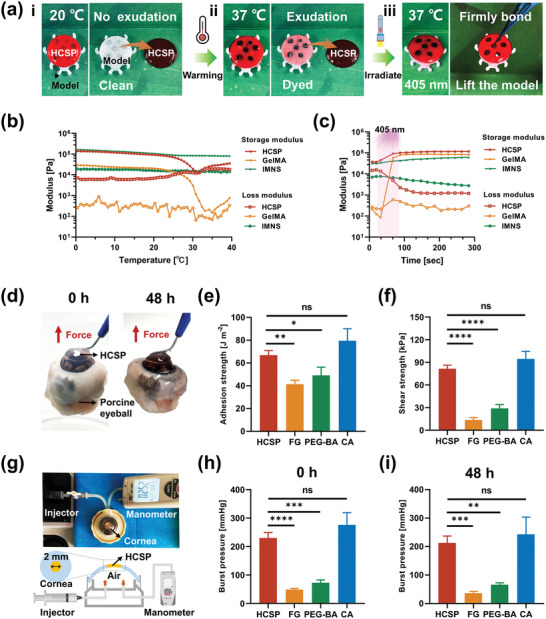
Tissue adhesion properties of HCSPs. a) Demonstration of temperature and light responsiveness of HCSPs. (i) At 20 °C, red dye‐labeled GelMA within HCSPs did not leach out to stain the model. (ii) At 37 °C, the model surface was stained by exuding GelMA. (iii) After 405 nm light irradiation, HCSPs firmly adhered to the model surface. Rheological properties of HCSPs, 20% GelMA, and IMNS b) at different test temperatures and c) before and after 405 nm light exposure. d) Images showing the adhesiveness of HCSPs to porcine eyeball. e) Adhesion and f) shear strength of HCSPs and commercial adhesives to corneal stroma. (FG: fibrin glue; PEG‐BA: PEG‐based adhesive; CA: cyanoacrylate adhesive). g) Illustration of the burst pressure measurements. Maximum IOP sustained by HCSPs and commercial adhesives at h) 0 h and i) 48 h. Data represented as mean ± SD. *, *p* < 0.05; **, *p* < 0.01; ***, *p* < 0.001; ****, *p* < 0.0001. ns, not significant.

An in vitro simulation experiment was performed to verify the environmental responsiveness of the HCSPs (**Figure** **4**a). To trace the adhesive, HCSPs containing red‐dye‐labeled GelMA were prepared. At 20 °C, no staining was observed on the model surfaces after the removal of the HCSPs. As the temperature increased to 37 °C, the HCSPs detached again, and the models became stained red. Following exposure to 405 nm light for 60 s, the models were lifted together with the HCSPs, indicating strong adhesion.

Rheological analysis further validated the temperature‐ and light‐responsiveness of HCSP. As the test temperature exceeded 25 °C, the curves of the storage and loss moduli of the HCSPs began to approach each other and tanδ increased, likely due to the exudation of liquid GelMA from the HCSP matrix (Figure 4b; Figure S7, Supporting Information). Upon exposure to 405 nm light at 37 °C, the storage‐to‐loss modulus ratio increased, reflecting the enhanced viscoelasticity of the HCSPs (Figure 4c).

To visualize the adhesion of the HCSPs to the natural corneal stroma, the HCSPs were applied to de‐epithelialized porcine corneal surfaces. After irradiation with 405 nm light, the porcine eyeballs were lifted along with the HCSPs, indicating that the interfacial bonding was sufficient to withstand the weight of the eyeball. The HCSPs maintained stable adhesion to the cornea even after 48 h (Figure 4d). The adhesive strength was assessed using a 180° peel test, and the results were compared with those of commercial medical adhesives including fibrin glue, polyethylene glycol (PEG)‐based adhesives, and cyanoacrylate adhesives (Figure S8a, Supporting Information). Quantitative results revealed that the adhesive strength of the HCSPs reached 67.8 ± 4.3 J m^−2^ (Figure 4e), significantly higher than those of fibrin glue (41.2 ± 3.7 J m^−2^; *P* = 0.005) and PEG‐based adhesives (48.9 ± 7.4 J m^−2^; *P* = 0.037). The shear strength of the HCSPs were investigated using a modified lap‐shear test (Figure S8b, Supporting Information). Similar to the results of the adhesive strength test, the HCSPs exhibited a shear strength of 81.6 ± 4.6 kPa, higher than that of fibrin glue (13.6 ± 3.1 kPa) and PEG‐based adhesives (29.0 ± 4.8 kPa) (Figure 4f). The adhesion properties of HCSPs were comparable to those of cyanoacrylate glue, a high‐strength tissue adhesive. However, cyanoacrylate glue is cytotoxic and can cause corneal opacity and neovascularization when applied to the ocular surface.^[^ ^25^ ^]^

Next, the sealing effect of HCSPs on corneal perforations was evaluated via burst pressure testing. Stromal defects (2 mm full thickness) were created at the center of the porcine corneas and mounted onto an artificial anterior chamber (Figure 4g). After repairing the perforations with HCSPs, the air was gradually injected into the chamber until the repaired corneas burst, and the maximum intraocular pressure (IOP) was recorded. The results showed that the HCSP‐sealed corneas withstood a peak IOP of 230.0 ± 19.3 mmHg (Figure 4h), significantly higher than that of the corneas sealed with fibrin glue (48.7 ± 4.0 mmHg; *P* = 0.0001) and PEG‐based adhesives (72.7 ± 10.0 mmHg; *P* = 0.0002). After 48 h of repair, the HCSPs continued to seal the perforations effectively, with a maximum IOP of 212.0 ± 25.2 mmHg (Figure 4i). The burst pressure of the HCSP‐sealed corneas was approximately ten‐fold higher than the physiological IOP (10–20 mmHg), indicating a high sealing potential of HCSPs against abrupt elevations in IOP, such as eye rubbing and squinting.

### 2.4 Cytocompatibility and Phenotype Regulation of HCSPs

**Figure 5:**
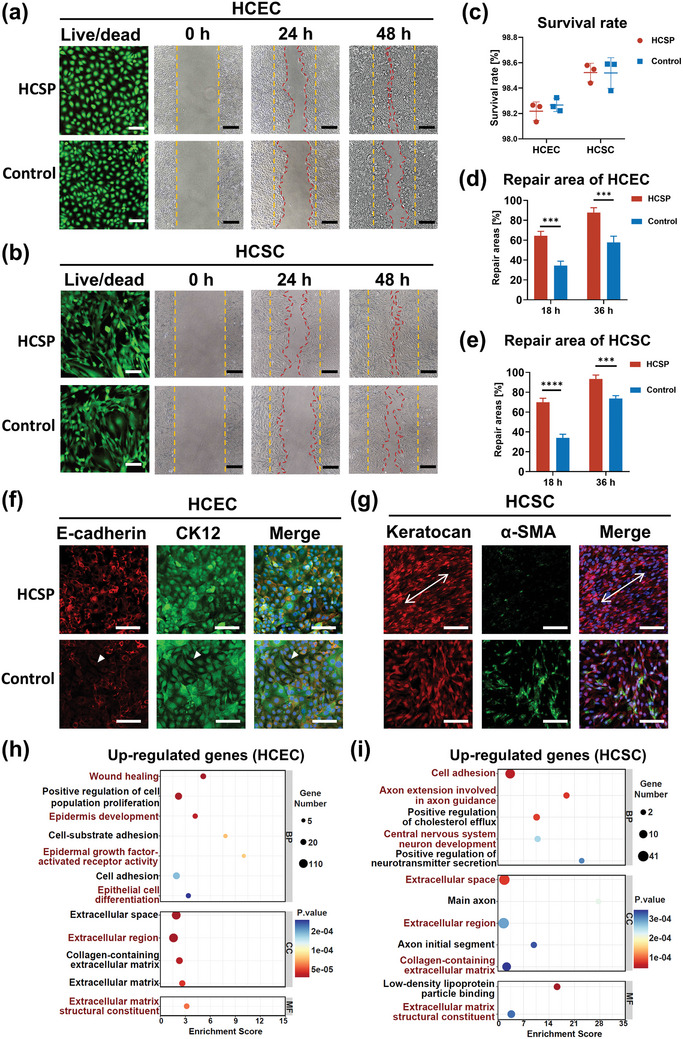
Cytocompatibility and phenotypic regulation of HCSPs. Representative live/dead fluorescence staining and scratch assay images of a) HCECs and b) HCSCs; scale bars = 100 µm for live/dead staining and 200 µm for scratch assay. c) Survival rate of HCECs and HCSCs. Percentages of repaired areas for d) HCECs and e) HCSCs at 18 and 36 h post scratch. Immunofluorescence images of f) HCECs and g) HCSCs cultured on HCSPs or directly on well plates; scale bars = 100 µm. GO enrichment analysis of upregulated genes in h) HCECs and i) HCSCs (cells cultured on HCSPs versus well plates). Data represented as mean ± SD. ***, *P *< 0.001; ****, *p* < 0.0001. ns, not significant.

Live/dead staining was performed to evaluate the cytocompatibility of the HCSPs. The results revealed that human corneal epithelial cells (HCECs) and human corneal stromal fibroblast cells (HCSCs) exhibited intense green fluorescence (live) and negligible red fluorescence (dead), with viability exceeding 95% (**Figure** **5**a–c). Monolayer cell scratch assays indicated an accelerated healing process in the HCSPs group, with a healing rate of above 80% within 36 h (Figure 5d,e).

Immunofluorescence staining was performed to investigate the ability of HCSPs to maintain the phenotypes of corneal epithelial and stromal cells. As shown in Figure 5f, HCECs cultured on HCSPs expressed the epithelial markers E‐cadherin and CK12, exhibiting a polygonal morphology with tight junctions. In contrast, some HCECs cultured directly on well plates exhibited potential epithelial‐to‐mesenchymal transition (EMT) with elongated cell morphologies and reduced E‐cadherin expression (indicated by white arrowheads in Figure 5f). The hCECM and natural topographical cues within HCSPs provided HCECs with a microenvironment similar to that in vivo, potentially reducing the incidence of EMT.^[^ ^26^ ^]^ The HCSCs cultured on the HCSPs expressed the keratocyte marker keratocan, with minimal expression of the myofibroblast marker α‐SMA (Figure 5g). Similarly, the HCSPs may influence the polarity of the HCSCs, resulting in regular alignment (indicated by white arrows in Figure 5g). These findings are consistent with recent reports that bionic‐structured hydrogels promote the ordered growth of keratocytes and inhibit their phenotypic transformation.^[^ ^5^, ^27^ ^]^

Whole‐transcriptome RNA sequencing was performed to evaluate the effects of HCSPs on corneal cells. Differential expression analysis revealed that 1067 and 493 genes were upregulated in the HCECs and HCSCs cultured on the HCSPs, respectively (Figure S9, Supporting Information). The gene ontology (GO) analysis results indicated that the upregulated genes in the HCECs were primarily enriched in terms of wound healing, epidermal development, and ECM constituents (Figure 5h). Terms associated with neurogenesis were enriched in the upregulated genes of HCSCs (Figure 5i). Keratocytes actively promote corneal nerve development by secreting neurotrophic factors,^[^ ^28^ ^]^ whereas myofibroblasts impede nerve regeneration.^[^ ^29^ ^]^ This finding was supported by the immunofluorescence staining results, which revealed that HCSPs maintained the HCSC phenotype and inhibited myofibroblastic differentiation.

### 2.5 In Vivo Application of HCSPs for Lamellar Keratoplasty

**Figure 6:**
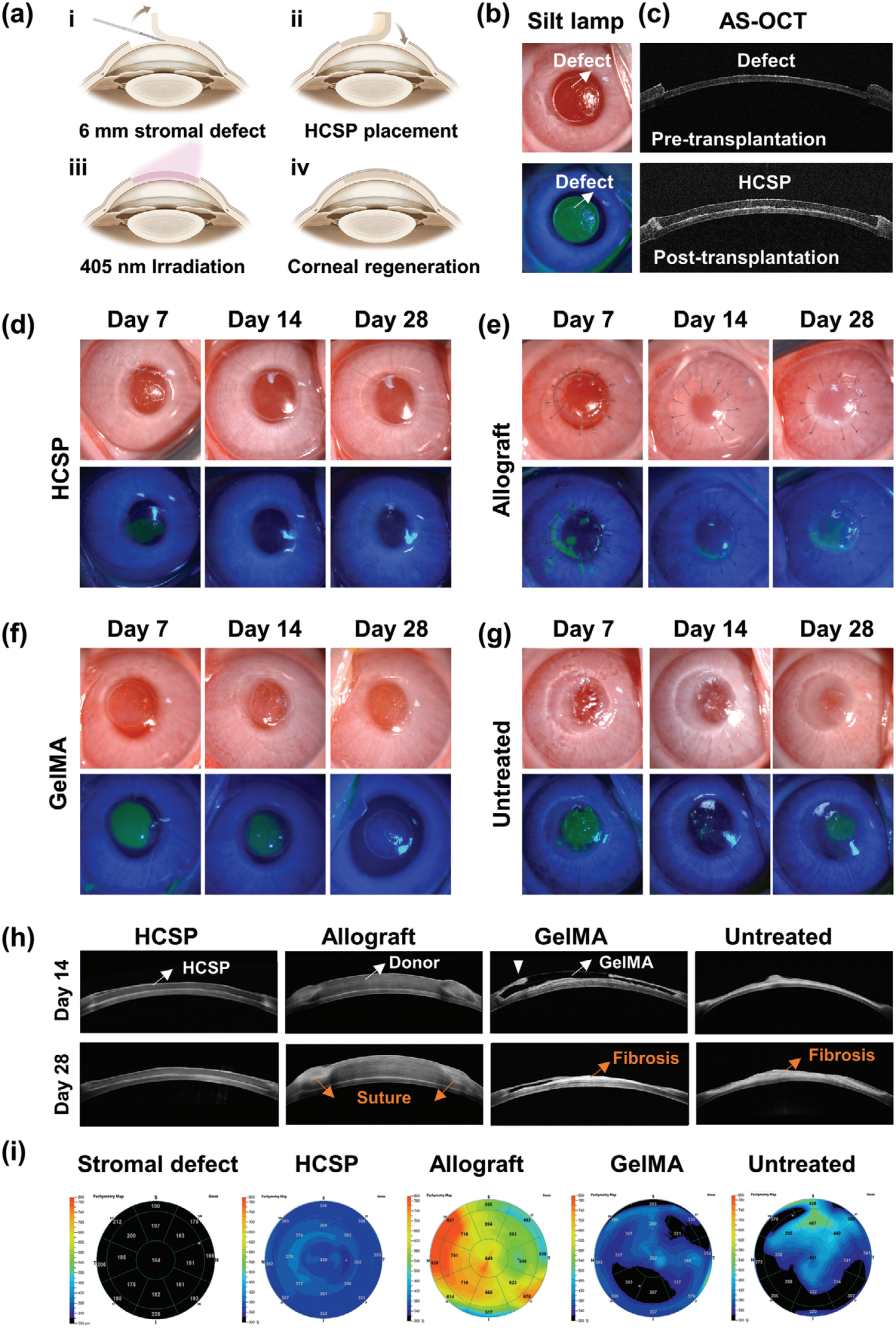
In vivo repair of lamellar stromal defects in rabbit cornea. a) Schematic of procedures for creating lamellar defects and repairing them with HCSPs. b) Slit lamp microscopy and fluorescence staining after stromal excision. c) AS‐OCT images pre‐ and post‐transplantation. Slit‐lamp microscopy and fluorescence staining images of d) HCSP‐, e) allograft‐, and f) 20% GelMA‐repaired groups; and g) untreated controls at 7, 14, and 28 d post‐transplantation. h) AS‐OCT images at 14 and 28 d post‐operation. i) Pachymetry maps obtained pre‐transplantation and at 28 d post‐transplantation.

To assess the in vivo repair efficacy of HCSPs, a lamellar stromal defect (6 mm in diameter; 200 µm in depth) was created in the rabbit cornea. As illustrated in **Figure** **6**a, the HCSPs were user‐friendly. After administering HCSPs to the recipient bed, the subsequent procedure required only positional adjustment of the implant and irradiation for 60 s. After transplantation, anterior segment optical coherence tomography (AS‐OCT) images showed that the stromal defect was repaired by the HCSP, resulting in a smooth surface (Figure 6c). Corneas that received allograft (suture group) or GelMA (sutureless group) transplantation, along with untreated corneas were used as control.

Slit‐lamp examination and fluorescein staining revealed that epithelial healing was achieved within 2 weeks in the HCSP group, and the cornea remained transparent without inflammation or neovascularization throughout the 28 d follow‐up (Figure 6d). In contrast, the allograft group experienced suture‐related complications in which an epithelial defect and a grey‐white ulcer were observed, possibly due to continuous ocular surface irritation caused by loose sutures (Figure 6e). GelMA‐repaired corneas revealed delayed epithelial healing and corneal haze (Figure 6f). GelMA was reported to have successfully repaired focal stomal defects (3 mm diameter, ≈9% corneal area). However, for a 6 mm lamellar defect, covering nearly 40% of the cornea, the hydrogel must meet higher demands in cohesiveness, anti‐degradation, restoration of curvature, and epithelial stability.^[^ ^13^ ^]^ A recent study, where GelMA was used to repair 4.5 mm defects in rabbit, showed similar results to our findings.^[^ ^11a^ ^]^ Thus, the size of the defect area may be a major factor influencing the wound healing of GelMA‐repaired corneas. In comparison to the treatment group, the untreated corneas exhibited scarred repair with a rough surface (Figure 6g).

The AS‐OCT findings further confirmed that the HCSPs remained intact without dissolution or delamination during the follow‐up period, exhibiting a reflectivity similar to that of the normal stroma (Figure 6h). The suture group exhibited graft edema and edge deformation owing to suture irritation and traction (Figure 6h). The GelMA‐treated corneas exhibited a steep repair surface, which likely hindered epithelial migration (indicated by the white arrowhead in Figure 6h). Furthermore, persistent epithelial nonhealing accelerated the degradation of the GelMA hydrogel, accompanied by matrix fibrosis. The pachymetry maps indicated more uniform thickness restoration in the HCSP‐repaired corneas than in the other treatment groups (Figure 6i).

**Figure 7:**
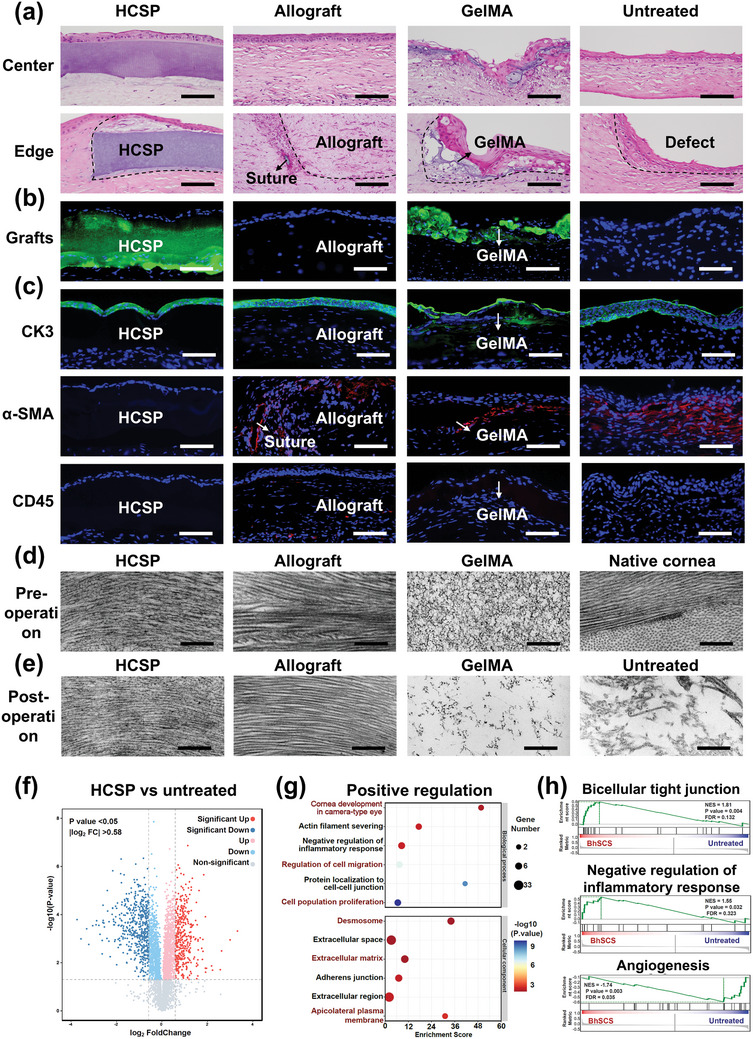
Histological, ultrastructural, and proteomic evaluations of repaired rabbit corneas. a) H&E staining for HCSP, allograft, and GelMA treated and untreated corneas at 28 d post‐operation; scale bars = 100 µm. b) Fluorescence staining for HCSP‐ and GelMA‐treated corneas at 28 d post‐operation; scale bars = 100 µm. c) Immunofluorescence for CK3, α‐SMA, and CD45 at 28 d post‐operation; scale bars = 100 µm. d) Pre‐implantation TEM images of HCSP, allograft, GelMA, and native cornea; scale bars = 500 nm. e) TEM images of repaired corneal stroma at 28 d post‐operation from various treatment groups; scale bars = 500 nm. f) Volcano plot, g) Significant GO terms for biological processes and cellular components of upregulated proteins, and h) GSEA plots of representative protein sets: bicellular tight junctions, negative regulation of inflammatory response, and angiogenesis (HCSP‐treated vs untreated corneas).

A histological evaluation was performed 28 d post‐operatively. H&E staining demonstrated good biointegration of the HCSPs with the recipient corneas in both the central and peripheral surgical areas (**Figure** **7**a). In contrast, fibrosis was observed around sutures in the allograft group. The GelMA group exhibited graft degradation, characterized by abundant matrix gaps, thickness loss, and compensatory epithelial hyperproliferation.

The implants were labeled with green fluorescence to visualize the HCSPs in vivo. As shown in Figure 7b, the HCSPs maintained their structural integrity with a thickness of ≈200 µm. In contrast, the GelMA matrices exhibited fragmentation. Immunofluorescence staining indicated that the surfaces of the HCSPs were covered with multilayered epithelial cells expressing the terminal differentiation marker CK3 without epithelial ingrowth or abnormal thickening (Figure 7c). Additionally, no intense α‐SMA expression was observed in the HCSP‐treated corneas, whereas those from the other treatment groups exhibited myofibroblast aggregation around the sutures and implants. CD45 staining indicated a minimal immune response to HCSPs (Figure 7c).

At 28 d post‐operatively, TEM images revealed that the HCSP‐repaired regions (Figure 7e) exhibited natural stroma‐like structures without notable changes compared to their pre‐implantation state (Figure 7d). In comparison, the polymer network of the GelMA hydrogel became sparse owing to its degradation (Figure 7e). The gradual degradation of HCSPs is expected to take several months to years, aligning with the stromal regeneration timeline.^[^ ^30^ ^]^ The degradation of PEGDA primarily results from the slow hydrolysis of the end‐group acrylate esters. Moreover, the oxidation of the ether backbone of PEGs also contributes to this process.^[^ ^31^ ^]^ Ultimately, the released small‐molecule PEGs and monomers diffuse out of the eye or enter the aqueous humor, where they are cleared through systemic metabolism.^[^ ^32^ ^]^

Proteomic analysis further validated the role of HCSPs in corneal injury repair. Compared with the untreated group, 887 differentially expressed proteins were identified in the HCSP‐treated group, with 385 upregulated and 502 downregulated proteins (Figure 7f), indicating that HCSPs extensively intervened in the healing process of stromal injuries. GO analysis revealed that the upregulated proteins were primarily enriched in biological processes associated with wound healing, including corneal development, cell migration, and proliferation (Figure 7g). Moreover, cellular component analysis revealed significant enrichment of ECM and cell adhesion. The gene set enrichment analysis (GSEA) results further confirmed that HCSPs promoted bicellular tight junctions and inhibited inflammation and neovascularization (Figure 7h).

### 2.6 In Vivo Repair of Corneal Microperforations with HCSPs

**Figure 8:**
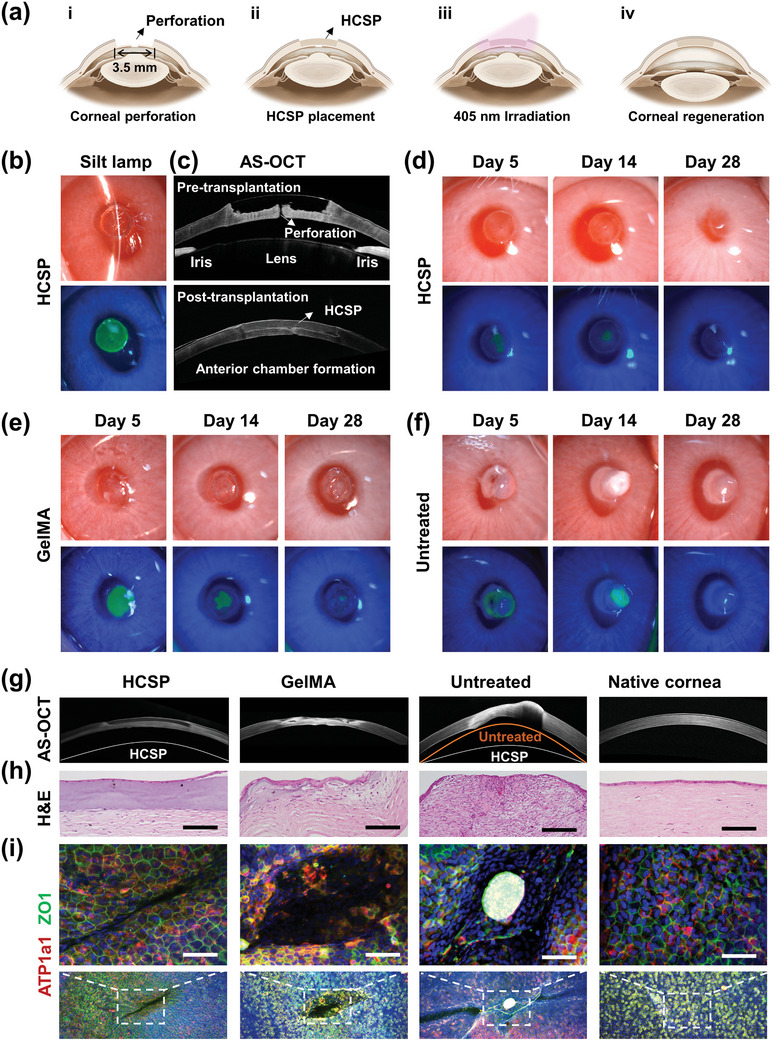
In vivo repairing of rabbit corneal microperforations. a) Schematic showing the creation of corneal perforation and repair using HCSPs. b) Slit‐lamp and fluorescence staining images recorded after corneal perforation. c) AS‐OCT images recorded before and after HCSP transplantation. Slit‐lamp and fluorescence staining images of d) HCSP‐ and e) GelMA‐repaired groups and f) untreated controls recorded at 5, 14, and 28 d post‐transplantation. g) AS‐OCT and h) H&E staining images of native corneas and those in different treatment groups at 28 d post‐operation; scale bars = 100 µm. i) Immunofluorescence staining images of endothelium in native cornea and different treatment groups at 28 d post‐operation; scale bars = 50 µm.

To evaluate the in vivo effectiveness of HCSPs in repairing microperforations, a model was developed to mimic corneal perforation injuries induced by iron debris during welding operations. First, a 3.5 mm diameter lamellar stroma was excised to simulate the debridement of burned or contaminated tissue in the perforation periphery. Subsequently, a perforation was created at the center of the lamellar defect using a 25 G needle. An HCSP graft of comparable size was then positioned onto the recipient bed and irradiated using 405 nm light to seal the perforation (**Figure** **8**a). Slit‐lamp and AS‐OCT observations performed after the perforations revealed eyeball deformation and anterior displacement of the ocular contents (Figure 8b,c). After the application of HCSP, the perforation was sealed, and the anterior chamber was restored (Figure 8c). In this study, GelMA‐treated and untreated corneas served as control groups.

A 28 d follow‐up revealed that HCSP‐treated eyeballs maintained the anterior chamber depth without aqueous leakage or synechiae. The repaired regions remained transparent, and epithelial healing was observed (Figure 8d). In contrast, GelMA‐treated corneas exhibited rough surfaces and stromal haze (Figure 8e), which may be attributed to the dilution and loss of the GelMA precursor caused by the active outflow of aqueous humor.^[^ ^33^ ^]^ The untreated group exhibited corneal ectasia and edema around the perforations (Figure 8f). The results of the AS‐OCT examination indicated that the HCSP‐repaired corneas withstood physiological IOP levels and re‐established a normal corneal curvature (Figure 8g).

H&E staining revealed that HCSP‐repaired corneas were covered by epithelial cells without evident infiltration by immune cells or fibroblasts (Figure 8h). Immunofluorescence staining was performed to evaluate the effects of HCSPs on corneal endothelial cells. In the HCSP‐treated corneas, the endothelial cells exhibited a hexagonal morphology and expressed tight junction protein‐1 (ZO‐1) and Na^+^/K^+^ ATPase (ATP1A1), which are critical for maintaining stromal hydration and transparency (Figure 8i).^[^ ^34^ ^]^ In addition, the HCSP‐repaired perforations had a neater closure than the GelMA‐repaired and untreated groups (Figure 8i). This improved closure could be attributed to the mechanical support provided to the corneal endothelium by HCSPs after perforation, which prevented the formation of pits and folds under IOP. These findings indicate that the HCSPs enabled the immediate and controlled repair of corneal microperforations while restoring intraocular homeostasis.

## 3 Conclusion

This study introduced an innovative strategy for preparing artificial corneal substitutes that replicate the natural stromal hierarchical structure and proteomic composition. The approach, free from reliance on advanced equipment and complex procedures, utilized tissue‐level bioassembly to enable HCSPs to precisely replicate the native stroma at the micro‐ and nanoscales while retaining the majority of the corneal ECM components. HCSPs exhibited optical properties comparable to those of natural corneas. Compared to traditional substitutes, hydrogel skeleton‐reinforced HCSPs demonstrated superior anti‐swelling, anti‐degradation, and tissue adhesion properties. Furthermore, in vitro studies confirmed that HCSPs promoted corneal epithelial and stromal cell survival and migration while preserving cell phenotypes. Clinical observations during large‐scale lamellar keratoplasty and corneal microperforation repair demonstrated that HCSPs were more advantageous than allograft transplantations in terms of simplified surgical procedures and reduced post‐operative complications. Furthermore, compared with in situ‐cured hydrogels, HCSPs rapidly accelerated wound healing and exhibited stability. In summary, HCSPs hold the potential to mitigate the shortage of donor corneas and provide valuable insights for the development of biomimetic biomaterials.

Although HCSPs demonstrated good biocompatibility and promoted wound healing 1‐month post‐corneal transplantation, their long‐term performance regarding material degradation and stromal regeneration remained unassessed. Furthermore, there remains a gap in mechanical properties between HCSPs and natural corneas. Future research should focus on incorporating tougher and stiffer hydrogel skeletons for the fabrication of HCSPs. In addition, long‐term in vivo studies should be conducted to prepare for future clinical trials.

## 4 Experimental Section

### Materials

Recombinant endonuclease was purchased from Sino Biological (Beijing, China). PEGDA (M_n_ = 700 Da) was purchased from Sigma‐Aldrich (MO, USA). GelMA (90% substitution) was purchased from EFL Tech (Suzhou, China). Phosphate‐buffered saline (PBS), 4% paraformaldehyde and 3% bovine serum albumin were purchased from SolarBio (Beijing, China). The porcine fibrin sealant kit was purchased from Johnson & Johnson (NJ, USA). PEG‐based glue was purchased from Success Bio‐tech (Shandong, China). Cyanoacrylate glue was purchased from Haoyuan (Shanghai, China). The live/dead double‐dye kit was purchased from Abbkine (GA, USA). Fetal bovine serum (FBS) was purchased from Hyclone (UT, USA). All other materials were purchased from Sigma‐Aldrich (MO, USA) unless otherwise specified.

### Decellularization of Human Corneal Lenticules

Corneal lenticules were extracted intraoperatively using femtosecond laser small‐incision lenticule extraction surgeries at the Eye Hospital of Shandong First Medical University. All surgeries were performed to correct myopia or myopic astigmatism. Informed consent was obtained from all patients. The procurement of human corneal tissue in this study strictly adhered to the tenets of the Ethics Committee of Shandong Eye Hospital (No. SDSYKYY202102‐1).

The extracted corneal lenticules were treated twice with high hydrostatic pressure (200 MPa for 2 min) and subsequently immersed in a decellularization solution containing 0.5% (w/v) sodium lauroylglutamate and recombinant endonuclease (500 U mL^−1^) at 50 mmHg colloid osmotic pressure for 2 h at 37 °C. Next, the samples were washed thoroughly with sterile 10 mm phosphate‐buffered saline (1 × PBS). Finally, the acellular lenticules were sterilized using Co‐60 irradiation (15 kGy).

### Preparation of hCECM

The acellular lenticules were lyophilized for 2 d and subsequently ground to a powder using a tissue fragmentation oscillator (Qiagen, Hilden, Germany). The pulverized lenticules were digested in a 1 mL pepsin/HCl solution (30 mg acellular lenticules were added to 3 mg pepsin in 0.1 m HCl) for 48 h at 37 °C. Subsequently, the solution was neutralized with 1 N NaOH and 100 mm PBS. After lyophilization for 3 d, the obtained hCECM was stored at −80 °C.

### Harvesting and Decellularization of Porcine Corneas

Fresh porcine eyes (4‐6‐month‐old Duroc swine, Pujiang Farm, Sichuan, China) were harvested within 2 h post‐mortem and washed with 1 × PBS containing tobramycin (1 mg mL^−1^). After mechanically removing the epithelium, the anterior corneal stroma (thickness: 400 µm for the in vitro study and 200 µm for the in vivo study; diameter: 9 mm) was excised using a femtosecond laser‐cutting machine (Wavelight FS200; Fort Worth, TX). The corneas were decellularized according to the protocol for human corneal lenticules.

### Preparation of IMNS

The DPCs were incubated in a PEGDA solution (0.2 g mL^−1^) containing 0.3% lithium phenyl‐2,4,6‐trimethybenzoylphosphinate (LAP) under a vacuum for 6 h, followed by shaking at 120 rpm for 12 h. The PEGDA solution on the DPC surface was blotted dry with lint‐free paper, placed on the upper surface of a corneal contact lens mold (Liangyu, Suzhou, China), and irradiated with 365 nm light (100 mW cm^−2^, EFL, Suzhou, China) for 1 min. The obtained gel‐tissue complex was then digested in a pepsin solution (30 mg mL^−1^) containing 0.1 N HCl and agitated at 120 rpm for 72 h at 37 °C. Finally, IMNSs were prepared by washing the digested sample six times with 1 × PBS for 30 min each.

### Assembly of HCSPs

The IMNSs were immersed in a 10% (w/v) hCECM solution, followed by shaking at 120 rpm for 24 h under vacuum. Subsequently, the IMNS were maintained at 4 °C for 30 min to solidify the hCECM solution within the matrix, followed by immersion in precooled *N*‐cyclohexyl‐*N*′‐(2‐morpholinethyl) carbodiimide metho‐*p*‐toluenesulfonate (50 mg mL^−1^) /*N*‐hydroxysuccinimide solution (12 mg mL^−1^) and cross‐linked for 12 h at 4 °C. After rinsing with 1 × PBS, the hCECM‐integrated IMNS was immersed in a 20% GelMA solution containing 0.25% LAP, agitated at 120 rpm for 6 h at 37 °C, and then stored at 4 °C.

### Ultrastructural Analysis

For cryo‐SEM, hydrogel and corneal samples (*n* = 3) were rapidly frozen in slush nitrogen and then fractured in a vacuum at 100 °C to expose a fresh fracture surface. Subsequently, the frozen samples were sublimated at −80 °C for 15 min, followed by platinum coating. Finally, the samples were transferred under a vacuum to a cryo‐SEM (SU3500, QuorumPP3010, Tokyo, Japan) and imaged at −175 °C and 10 kV using backscattered electron signals.

For TEM, the samples (*n* = 3) were fixed with 2.5% (v/v) phosphate‐buffered glutaraldehyde at 4 °C for 4 h and post‐fixed with 1% (w/v) osmium tetroxide at 4 °C for 2 h. The samples were dehydrated using a graded series of acetone solutions. The samples were then embedded in Epon‐812 resin and cured at 60 °C for 24 h. Thin sections (60 nm) of the samples were obtained using an ultramicrotome (UC6; Leica, Wetzlar, Germany). Finally, the sections were contrast‐stained with 2% (w/v) aqueous uranyl acetate for 15 min and 1% (w/v) aqueous lead citrate for 10 min. JEM‐1400 (JEOL, Tokyo, Japan) was used for TEM, and images were captured using an Olympus iTEM 5.0 (Tokyo, Japan). All reagents mentioned in this subsection were purchased from Head Biotechnology, Beijing, China.

### Macroscopic Transparency and Light Transmittance

The samples were placed on prints and photographed from above under natural light for macroscopic transparency. To evaluate light transmittance, the samples (*n* = 3) were placed in cuvettes, and the absorbance of light (300–800 nm) was measured using an ultraviolet‐visible spectrophotometer (Molecular Devices, Silicon Valley, CA). Transmittance analysis was conducted using SoftMax Pro version 4.8 (Molecular Devices, Silicon Valley, CA).

### Swelling Ratio

The initial weights (W_0_) of the samples were recorded using an electronic balance (*n* = 3). The samples were then immersed in artificial tears at 37 °C for 7 d and weighed at predetermined time points each day (W_t_). The artificial tear formulation consisted of a complex salt solution (Table S1, Supporting Information), human lysozyme (1.8 mg mL^−1^), and human lactoferrin (1.9 mg mL^−1^). The swelling ratio was calculated using Equation (1).

(1)

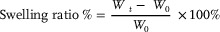




### Enzyme Resistance Assay

In vitro degradability was evaluated using collagenase from *Clostridium histolyticum* (Sigma‐Aldrich). After the initial weights (*W_0_*) were recorded, the samples (*n* = 3) were incubated in collagenase solution (100 U mL^−1^) at 37 °C for 7 d. The samples were weighed at predetermined times (*W_t_*) and the residual masses were calculated using Equation (2).

(2) $$\mathrm{Residual}\;\mathrm{mass}\;\%=\frac{W_{t}}{W_{0}}\;\times \;100\%$$

### Tensile Strength

Tensile tests were conducted using a BioTester 5000 system (CellScale, Waterloo, Canada) with biaxial piezoelectric elements. The samples (*n* = 3) were mounted on a tissue tester using BioRakes (CellScale, Waterloo, Canada). The samples were then immersed in 1 × PBS and stretched at a rate of 0.02 mm s^−1^. LabJoy version 9.05 (CellScale, Waterloo, Canada) was used for the data analysis.

### Rheological Studies

Rheological analyses were performed using an ARES‐G2 rheometer (TA Instruments). The HCSPs (*n* = 3) were subjected to an oscillatory load at 0.25% strain and a constant frequency of 6.28 rad s⁻¹ within the linear viscoelastic region. IMNS and GelMA served as controls (*n* = 3 per group). In the dynamic temperature ramp mode, the HCSPs were heated from 0 to 40 °C at a rate of 5 °C min^−1^. Rheological dynamic time‐sweep measurements of the samples were performed at 37 °C. After 30 s into the test, the samples were irradiated with 405 nm light (25 mW cm^−2^, EFL, Suzhou, China) for 60 s.

### In Vitro Adhesion Assay

The corneal epithelium was removed from freshly isolated porcine eyeballs (*n* = 3) using an epithelial scraper. The HCSPs were then applied to the pre‐warmed (37 °C) corneal stroma surfaces, incubated for 10 s, and irradiated with 405 nm light for 60 s. A tunnel knife was bonded to the HCSP surface using superglue (Krazy Glue, Atlanta, GA). Finally, adhesion between the HCSPs and porcine eyeballs was assessed by lifting a tunnel knife.

### Shear and Adhesion Strength Test

Freshly isolated porcine eyes were rinsed with 1 × PBS, and anterior lamellar caps (9 mm in diameter) were removed using a microkeratome system equipped with a 400‐micron head (Evolution 3E Console, Moria Surgical, France). The HCSPs (*n* = 3) were then transplanted to seal the stromal defects and irradiated with 405 nm light for 1 min at 37 °C. Porcine fibrin sealant, PEG‐based adhesive, and cyanoacrylate adhesive were used separately as control materials according to the manufacturer's instructions (*n* = 3 for each group). Next, the repaired corneas, including the limbus, were dissected into rectangular strips (15 mm in length and 5 mm in width). Poly (methyl methacrylate) films with a thickness of 50 µm were attached to rectangular strips. The films provided backing support and facilitated gripping for the HCSPs and control materials. Shear and adhesion strength tests were performed using a dynamic mechanical analyzer (Q800, TA Instruments, New Castle, DE) at a peeling speed of 5 mm min^−1^. To measure the shear strength, the samples were subjected to a modified lap‐shear test (American Society for Testing Materials, ASTM F2255). Shear strength was determined by dividing the maximum force by the total adhesive area. To measure adhesive strength, the prepared samples were subjected to a modified 180° peel test according to ASTM F2256, with the adhesive strength determined by dividing the plateau force by the width of the sample.

### Ex Vivo Burst Tests

Cylindrical perforations (2 mm in diameter) were created at the center of the corneas using a trephine. The HCSPs (2.25 mm in diameter; *n* = 3) were then transplanted to seal the perforations and irradiated with 405 nm light for 60 s at 37 °C. Commercial sealants were used as control materials (*n* = 3 in each group). Burst tests were performed immediately and after 48 h (the samples were stored at 4 °C). The repaired corneas were mounted on an artificial anterior chamber (Moria, Paris, France) connected to a syringe pump (LongerPump, Hebei, China) and pressure monitor (MedLab‐U/4C501H, Shanghai, China). Air was continuously injected into the artificial anterior chamber at 2.5 µL s^−1^ until the repaired cornea burst, and the maximum pressures were recorded.

### Proteomic Analysis

Frozen samples (*n* = 3) were ground into powder in liquid nitrogen. The protein lysate was placed in an ice bath for 30 min and ultrasonicated for 2 min on ice. After centrifugation at 4 °C for 20 min, the supernatants were collected. The protein concentrations in the supernatants were quantified using a bicinchoninic acid assay, and equal amounts of protein from each sample were digested with trypsin following a standard procedure.^[^ ^11e^ ^]^ Analyses were performed using a Tims TOF Pro mass spectrometer (Bruker, Billerica, MA, USA) with an electrospray source. The samples were loaded onto a C18 column (25 cm × 75 µm) on an EASY‐nLCTM 1200 system (Thermo Fisher Scientific, Waltham, MA, USA). Liquid chromatography‐tandem mass spectrometry spectra were searched using MaxQuant against the *Homo sapiens* UniProt database (2022.02) for component analysis and the *Oryctolagus* UniProt database (2023.02) for animal experiments. The differentially expressed proteins, identified with |log_2_ fold change| > 0.58 and *p *< 0.05, were subjected to GO analysis. GSEA was performed using GSEA v.3.0 (Broad Institute, Boston, MA) and gene signatures from MSigDB v.6.1.

### Cell Culture and Live/Dead Staining

The simian virus 40‐immortalized HCEC line was obtained from the American Type Culture Collection, CRL‐11135, Manassas, VA, USA) and cultured in Dulbecco's modified Eagle's medium/nutrient mixture F‐12 (DMEM/F12) with 10% FBS in a humidified atmosphere of 5% CO_2_ at 37 °C. The telomerase‐immortalized HCSC line provided by Dr. Jester was maintained in DMEM/F12 medium supplemented with 10% FBS.^[^ ^35^ ^]^

HCECs and HCSCs were seeded into 12‐well plates (1 × 10^5^ cells per well) and incubated at 37 °C with 5% CO_2_ for 12 h. The HCSPs were immersed in DMEM/F12 supplemented with 10% FBS for 24 h to prepare the leach liquors. After adding the leach liquors to the wells, the cells were incubated for another 36 h. Cells cultured directly in DMEM/F12 supplemented with 10% FBS were used as the control group. The cells were stained using a live/dead double‐dye kit according to the manufacturer's instructions. Images were recorded using a phase‐contrast microscope (Nikon Eclipse Ti‐U, Tokyo, Japan), and the number of living cells was counted using Photoshop CS (Adobe, San Jose, CA). Independent experiments were repeated at least three times.

### Cell Migration Assay

To evaluate the direct effect of the main HCSP components on cell migration, a mixed solution containing PEGDA (0.2 g mL^−1^), 0.3% (w/v) LAP, and 10% (w/v) hCECM was prepared. The mixed solution (250 µL) and 20% (w/v) GelMA solution (250 µL) were added to six‐well plates and incubated for 1 h at 37 °C. The coated wells were washed with cold 1 × PBS and illuminated for 60 s using 405 nm light. HCECs and HCSCs were seeded into pre‐coated six‐well plates (5 × 10^5^ cells well^−1^) and cultured until ≈80% confluence. Cells cultured on uncoated plates were used as controls. Scratches were created on the cell monolayer using sterile pipette tips, followed by 1 × PBS washing. Subsequently, the cells were cultured in DMEM/F12 supplemented with 10% FBS. Three images of scratches at different positions were captured using a phase‐contrast microscope at 0, 18, and 36 h post‐scratch. The scratched areas were quantified using ImageJ (National Institutes of Health, Bethesda, MD, USA). Independent experiments were repeated at least three times.

### Histological Staining

H&E staining was performed according to the manufacturer's instructions (Vector Laboratories). Immunofluorescence assays were performed according to the standard procedures. The samples were fixed with 4% paraformaldehyde, blocked with 3% bovine serum albumin for 1 h, and incubated overnight with primary antibodies. The details of all antibodies used in this study are provided in Table S2 (Supporting Information). The samples were subsequently washed with 1 × PBS and incubated with secondary antibodies for 1 h at room temperature (Table S2, Supporting Information). To evaluate the effect of HCSPs on cell phenotype in vitro, HCSPs were anchored to a 12‐well plate containing 1% (w/v) agar gel. Next, HCECs and HCSCs (1 × 10^5^ cells) were individually seeded onto the surfaces of the HCSPs and cultured for 7 d. Cells cultured directly in the well plate served as a control group. For in vivo experiments, corneal tissues were harvested and sectioned using a freezing microtome (Thermo Fisher Scientific). Green fluorescein‐labeled GelMA was used to visualize the material localization. The samples were observed using the snap mode of a confocal microscope (LSM 880; Carl Zeiss, Oberkochen, Germany). Endothelial staining was performed in tile‐scan mode using the confocal microscope.

### RNA Sequencing

The HCECs and HCSCs (1 × 10^5^ cells) were seeded onto the surfaces of HCSPs (*n *= 3) and cultured for 7 d. Cells cultured directly on the well plate served as the control group. Total RNA was extracted using TRIzol reagent (Invitrogen) and assessed for purity and quantity using a NanoDrop 2000 spectrophotometer (Thermo Fisher Scientific). For RNA sequencing, libraries were constructed using the Universal V6 RNA‐seq Library Prep Kit (Vazume, Nanjing, China) according to the manufacturer's instructions. Libraries were processed on an Illumina HiSeq X Ten platform using Trimmomatic for trimming and HISAT2 for mapping to the human genome (GRCh38). Differential expression was analyzed using the DESeq2 R package to identify genes with |log_2_ fold change | > 1 and q < 0.05. GO analyses were performed using the clusterProfiler R package.

### In Vivo Repair of Rabbit Corneal Defect

Healthy male New Zealand White rabbits (5–6 months old; Xilingjiao, Jinan, China) were used in this study. All animal experiments were conducted in accordance with the Association for Research in Vision and Ophthalmology Statement for the Use of Animals in Ophthalmic and Vision Research and approved by the Ethics Committee of the Eye Institute of Shandong First Medical University (No. SDSYKYJS 20 210 217). Rabbits were anesthetized intramuscularly with ketamine (40 mg kg^−1^) and chlorpromazine (20 mg kg^−1^).

Lamellar keratectomies were performed using a 6.0‐mm surgical trephine to a depth of 200 µm with intraoperative anterior segment optical coherence tomography (AS‐OCT, Optovue, Fremont, CA). HCSPs (*n* = 5) with a diameter of 6 mm were placed on the recipient bed and adjusted accordingly. After holding for 10 s, the grafts were irradiated with 405 nm light for 1 min. The control group included allogeneic transplantation, 20% GelMA repair, and non‐transplantation groups (in each group, *n* = 5). In the allogeneic transplantation group, the allografts (6.25 mm in diameter) were secured using 12 10‐0 nylon‐interrupted sutures (Kabushiki Kaisha, Tokyo, Japan). In the GelMA repair group, a 20% GelMA solution supplemented with 0.25% LAP was injected into the stromal defect and cured with 405 nm light for 1 min. Post‐operative medications included tobramycin and dexamethasone eye ointment (Société Anonyme (SA) Alcon‐Couvreur Naamloze Vennootschap (NV), Puurs, Belgium), which were initially administered twice daily for 5 d and later replaced with tobramycin and dexamethasone eye drops (SA Alcon‐Couvreur NV) for 14 d post‐operative.

For the creation of the perforation models, anterior lamellar keratectomies were performed using a 3.5 mm surgical trephine at the corneal center to a 200–250 µm depth. Corneal perforations were subsequently created by penetration using a 25 G needle at the center of the keratectomy area. The HCSPs (3.5 mm in diameter; *n* = 5) were applied to the defect and irradiated with 405 nm light for 1 min. The control group consisted of 20% GelMA‐repaired and non‐transplanted groups (*n* = 5 per group). In the GelMA repair group, after draining and drying the fluid from the anterior chamber, the defect was filled with a 20% GelMA solution and cured with 405 nm light for 1 min. Postoperative medications were the same as those described for lamellar keratectomies.

### Follow‐Up Examination

Clinical follow‐ups were performed using a slit‐lamp biomicroscope (KangHua, Chongqing, China), fluorescein staining, and AS‐OCT for 28 d. Specifically, on 7, 14, and 28 d post‐operative, the corneal clarity, neovascularization, inflammation, and infection were evaluated using slit lamps. Corneal epithelial integrity was evaluated using fluorescein sodium staining. Corneal cross sectional images and thicknesses were assessed using AS‐OCT.

### Statistical Analysis

Statistical analyses were performed using GraphPad Prism version 8.3.0 (San Diego, CA, USA). Data were presented as the mean ± standard deviation. Experiments with two groups were analyzed using Student's t‐test. Experiments with more than two groups were analyzed using one‐way analysis of variance and Tukey's post‐hoc test. The sample size (*n*) for each test is described in this section. Each experiment was performed at least thrice. All tests were two sided. Statistical significance was set at *p *< 0.05.

## Conflict of Interest

The authors declare no conflict of interest.

## Supporting information



Supporting Information

## Data Availability

The data that support the findings of this study are available from the corresponding author upon reasonable request.
