# Supplementary material for: Artificial Cornea Substitute Based on Hydrogel Skeletons with Natural Stromal Hierarchical Structure and Extracellular Matrix for Sutureless Transplantation
Source: Adv Sci (Weinh). 2025 Jan 24;12(19):2411540. doi: 10.1002/advs.202411540 (PMC12097023; doi:10.1002/advs.202411540)
Supplement: Supplementary file 1 — Supporting Information [file ADVS-12-2411540-s001.docx]

**Supplementary Information**

**Artificial Cornea Substitute Based on Hydrogel Skeletons with Natural Stromal Hierarchical Structure and Extracellular Matrix for Sutureless Transplantation**

*Long Zhao, Zhen Shi, Xiaoyu Zhang, Jingting Wang, Shang Yang, Fuyan Wang, Tan Li, Qingjun Zhou*, Ting Wang*, Weiyun Shi**

**This file includes**:

1. Experimental Section

2. Figure S1 to S9

3. Table S1 to S3

4. References

**1.** **Experimental Section**

*Preparation of Control Materials:* The RHCPs were prepared according to the protocol previously reported.^[1]^ A 13.7% (w/v) clinical-grade recombinant human collagen (Jinbo, Shanxi, China) solution was mixed with 1% (w/v) 1-ethyl-3-(3-dimethylaminopropyl) carbodiimide (EDC) and NHS. The mixture was immediately dispensed into contact lens molds (thickness: 400 μm; diameter: 9 mm) and crosslinked at 100% humidity at 21 °C for 24 h and then at 37 °C for another 24 h. Finally, the RHCPs were obtained and washed thoroughly with 1X PBS three times for 10 min each.

*Preparation of* *GelMA:* GelMA was synthesized according to a previously published protocol.^[2]^ A 10% (w/v) gelatin solution was prepared by dissolving gelatin (Sigma Aldrich, St. Louis, MO) in 1X PBS, followed by heating at 60 °C for 1 h. Next, 8% (v/v) methacrylic anhydride was added dropwise to the gelatin solution under continuous stirring at 50 °C for 3 h. The solution was then diluted with 1X PBS and dialyzed against deionized water at 50 °C for 5 d. After sterile filtration and lyophilization for 3 d, the obtained GelMA was stored at −80 °C. The methacrylation degree of three different GelMA samples was quantified using the 2,4,6-trinitrobenzene sulfonic acid (TNBS) assay, respectively.^[3]^ The results showed that the degree of methacrylation for GelMA was 89.2 ± 2.5% (Table S3, Supporting Information).

*Collagen Detection:* For the visualization of collagen fibers, samples (n = 3) were fixed in a 4% (v/v) formaldehyde overnight, followed by dehydration, paraffin embedding, and slicing into 4 μm thick sections. Masson’s trichrome staining was performed according to the manufacturer’s instructions (Masson's Trichrome Stain Kit, Solarbio, Beijing, China). Images were acquired using a phase-contrast microscope (Nikon Eclipse Ti-U, Tokyo, Japan).

For the quantification of collagen content, the samples (n = 3) were treated in pepsin solution (1 mg mL^-1^ in 0.05 M acetic acid) at 4 °C for 48 h. Soluble collagen was determined by Sirius red method and quantified against the bovine collagen standard curve (Sirius Red Total Collagen Detection Kit, Chondrex, Woodinville, WA).

*Proportion of primary component in HCSP*: Two sets of samples were prepared to calculate the ratio of the dry weight to wet weight of IMNS (R_i_) and the ratio of the dry weight of hCECM to the wet weight of integrated IMNS-hCECM (R_h_). The weights of the test samples during preparation were recorded: the wet weight of IMNS (W_i_), the wet weight of IMNS-hCECM (W_h_), the wet weight of HCSP (W_g_), and the dry weight of HCSP (W_g0_). The mass proportions of PEGDA, hCECM, and GelMA were calculated using the following equations.

$\text{Mass ratio of PEGDA \% = }\frac{\boldsymbol{W}_{\boldsymbol{i}}\boldsymbol{\times}\boldsymbol{R}_{\boldsymbol{i}}}{\text{W}_{\text{g}}}\text{×100\%}$

$\text{Mass ratio of }\text{hCECM}\text{ \% = }\frac{\boldsymbol{W}_{\boldsymbol{h}}\text{×}\boldsymbol{R}_{\boldsymbol{h}}\text{ }}{\text{W}_{\text{g}}}\text{×100\%}$

$\text{Mass ratio of }\text{GelMA}\text{ \% = }\frac{\text{(}\boldsymbol{W}_{\boldsymbol{g}\mathbf{0}}\boldsymbol{-}\text{ }\boldsymbol{W}_{\boldsymbol{i}}\boldsymbol{\times}\boldsymbol{R}_{\boldsymbol{i}}\boldsymbol{-}\boldsymbol{W}_{\boldsymbol{h}}\text{×}\boldsymbol{R}_{\boldsymbol{h}}\text{) }}{\text{W}_{\text{g}}}\text{×100\%}$

**2. Supplementary Figures**


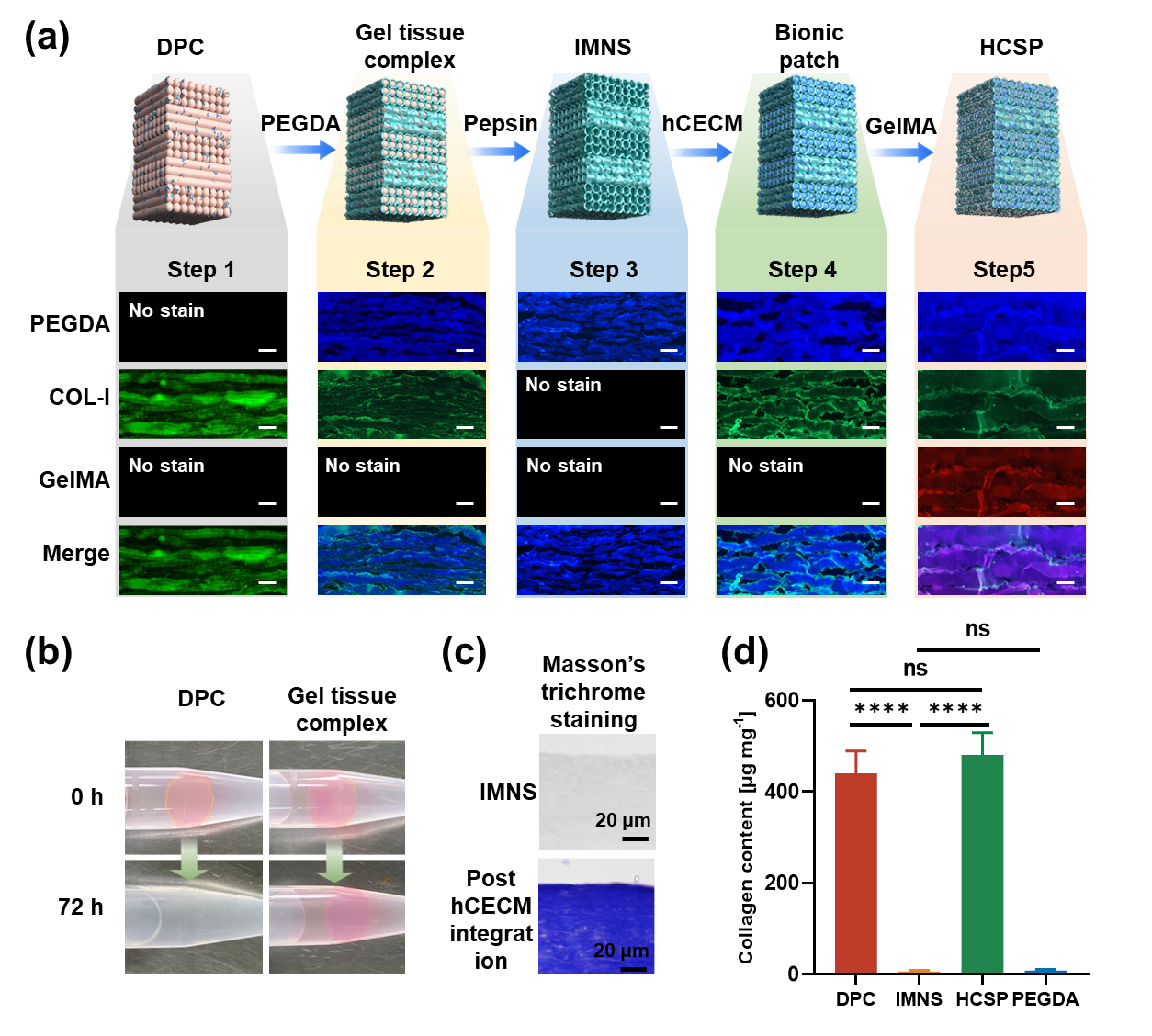


**Figure S1.** Histological, morphological, and protein content changes during HCSP construction. (a) Fluorescence staining showing the primary component changes during the stepwise construction of HCSP. The decellularized cornea (DPC) matrix exhibited collagen type I (COL-I) expression. After blue fluorescent-labeled PEGDA permeation, a uniform distribution within the DPC matrix was observed. Following pepsin treatment, the expression of COL-I in the corneal stroma disappeared, and the PEGDA retained. After perfusion with hCECM, extensive expression of COL-I reappeared within the IMNS. Finally, the red fluorescent-labeled GelMA infiltrated and distributed uniformly within HCSP; scale bars = 50 μm. (b) Comparison of DPC and gel-tissue composites following digestion with pepsin solution (30 mg ml^-1^) in 0.1N HCl for 0 and 72 h. DPCs were completely digested after 72 h, whereas the gel-tissue composite preserved its structure in pepsin solution. For visualization, samples were labeled with a red dye. (c) Representative Masson's trichrome staining images. Results indicated no collagen fiber staining in IMNS. After hCECM integration, blue-stained collagen were extensively distributed within the IMNS. (d) Total collagen content in DPC, IMNS, HCSP and PEGDA. Data represented as mean value ± SD. ****, *P* < 0.0001. ns, not significant.


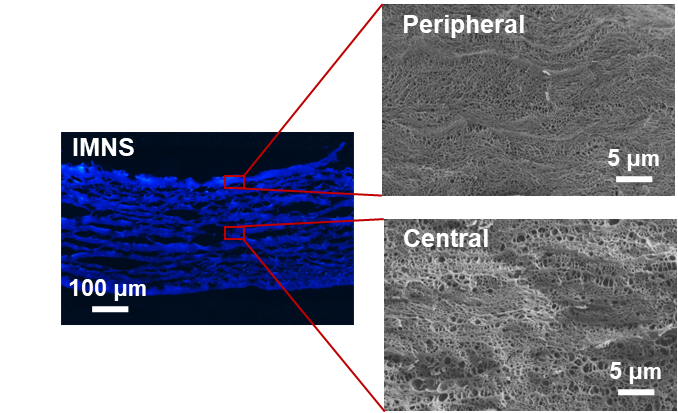


**Figure S2.** Fluorescence staining and scanning electron microscopy images of IMNS.


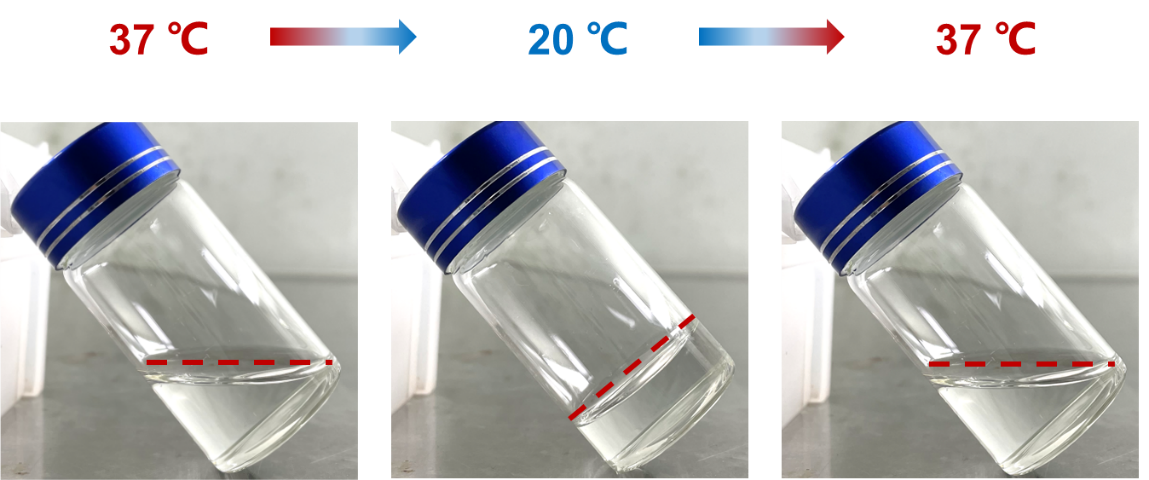


**Figure S3.** Reversible physical state transitions of 20% GelMA at different environmental temperatures. At 20°C, GelMA is in a gel state; at 37°C, GelMA transitions to a freely flowing liquid.


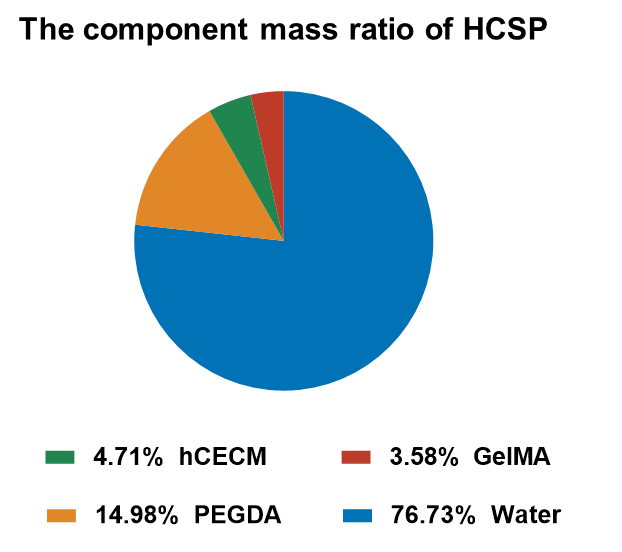


**Figure S4.** The mass proportion of hCECM, GelMA, PEGDA, and water in HCSP.


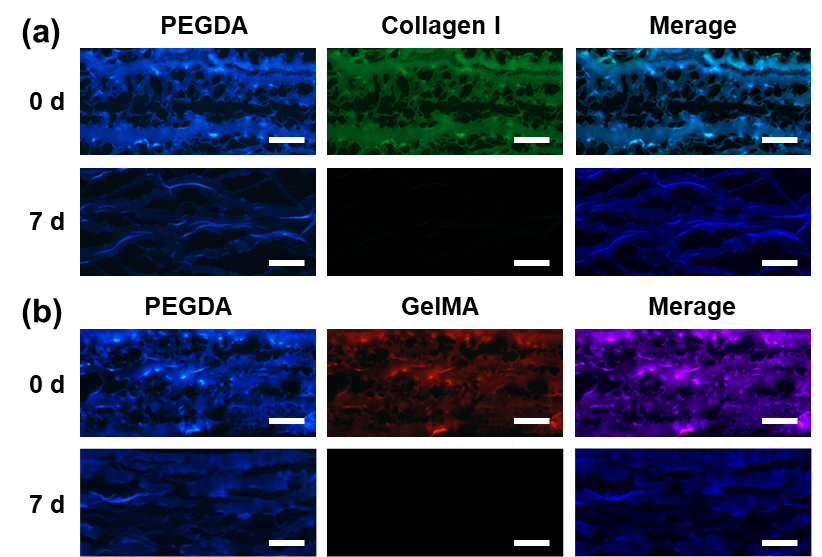


**Figure S5.** Fluorescence images before and after collagenase degradation. (a) hCECM integrated with IMNS, and (b) GelMA integrated with IMNS before and after 7 d of collagenase treatment. Blue fluorescence-labeled PEGDA and red fluorescence-labeled GelMA were used for visualization. Green fluorescence-labeled anti-collagen I antibody was applied to visualize hCECM. Scale bars = 100 µm.


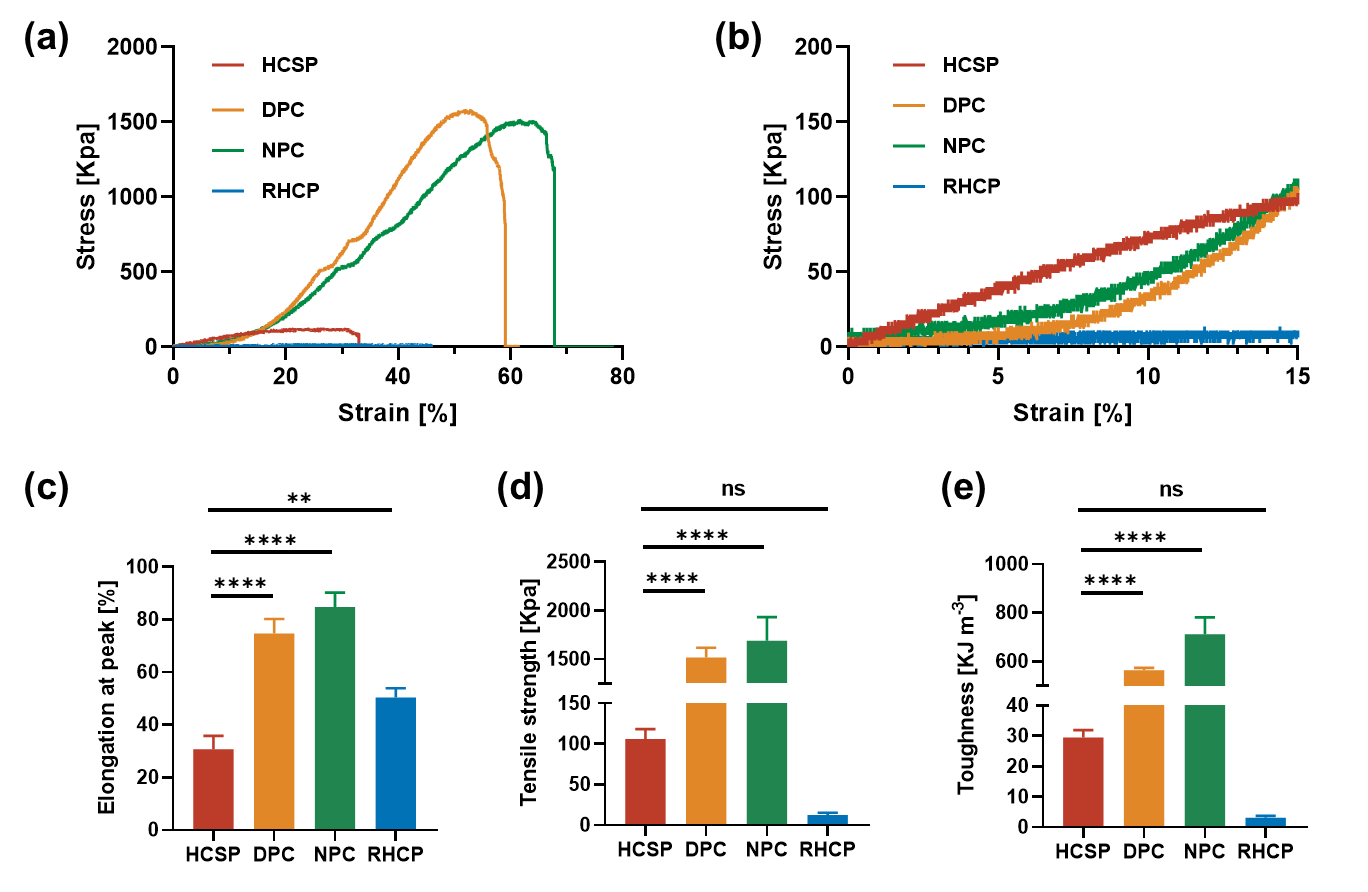


**Figure S6.** Biomechanical characteristics of HCSP. (a) Representative complete stress-strain curve for HCSP, DPC, NPC, and RHCP. DPC, decellularized porcine cornea; NPC, natural porcine cornea; RHCP, recombinant human collagen patch. (b) Stress variation within the 15% strain for representative samples. (c) Elongation at break, (d) Tensile strength, (e) Toughness of HCSP compared with DPC, NPC, and RHCP. Data represented as mean ± SD. **, P < 0.01; ****, P < 0.0001. ns, not significant.

**Figure S7.** Tanδ-temperature curve of HCSP, 20% GelMA prepolymer, and IMNS.


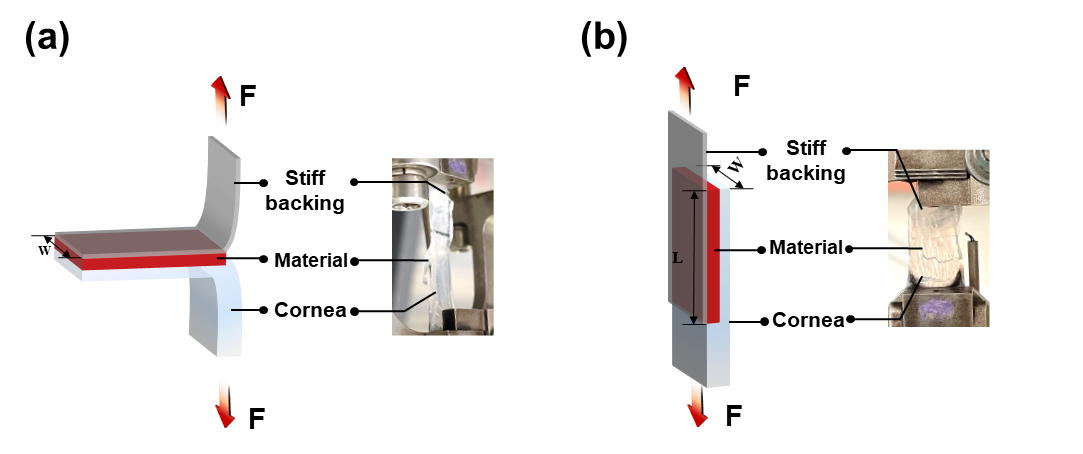


**Figure S8.** Setups for adhesion testing. (a) Setup for measurement of adhesion strength, based on the modified 180-degree peel test (ASTM F2256). (b) Setup for measurement of shear strength, based on the modified lap-shear test (ASTM F2255).


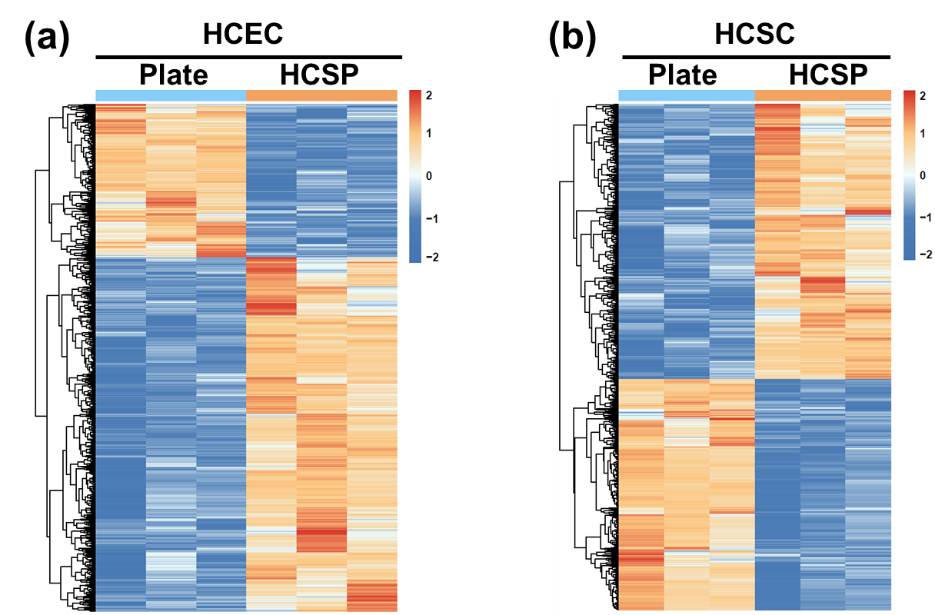


**Figure S9.** Heatmap of differentially expressed genes in (a) HCECs and (b) HCSCs after cluster analysis (Plate vs HCSP).

**3. Supplementary Tables**

**Table S1.** Complex salt components of artificial tears.

| Salt component | Molecular formula | | Concentration [mmol L^-1^] | |
| --- | --- | --- | --- | --- |
| Sodium chloride | | NaCl | | 90.0 |
| Potassium chloride | | KCl | | 16.0 |
| Sodium citrate | | Na_3_C_6_H_5_O_7_ | | 1.5 |
| Glucose | | C_6_H_12_O_6_ | | 0.2 |
| Urea | | CO(NH_2_)_2_ | | 1.2 |
| Calcium chloride | | CaCl_2_ | | 0.5 |
| Sodium carbonate | | Na_2_CO_3_ | | 12.0 |
| Potassium hydrogen carbonate | | KHCO_3_ | | 3.0 |
| Sodium phosphate dibasic | | Na_2_HPO_4_ | | 24.0 |

**Table S2.** Antibody information.

| Antibodies | Companies | Item No. | Dilution Rate |
| --- | --- | --- | --- |
| ATP1a1 | Biolegend | 3141704 | 1:100 |
| α-SMA | Abcam | ab7817 | 1:400 |
| Collagen I | Abcam | ab23446 | 1:200 |
| CK3 | Abcam | ab68260 | 1:200 |
| CK12 | Abcam | ab185627 | 1:50 |
| CD45 | Abcam | ab8216 | 1:100 |
| Keratocan | Abcam | ab113115 | 1:100 |
| E-cadherin | Abcam | ab231303 | 1:100 |
| ZO-1 | Abcam | ab216880 | 1:100 |
| Alexa Fluor 594 anti-rabbit | Invitrogen | A21270 | 1:500 |
| Alexa Fluor 488 anti-rabbit | Invitrogen | A11008 | 1:500 |
| Alexa Fluor 594 anti-mouse | Invitrogen | A21203 | 1:500 |
| Alexa Fluor 488 anti-mouse | Invitrogen | A21202 | 1:500 |

**Table S3.** Methacrylation Degree of GelMA

| Samples | Amount of methacryloyl [mmol g^-1^] | | Degree of substitution [%] | |
| --- | --- | --- | --- | --- |
| GelMA-1 | | 0.278 | | 86.4 |
| GelMA-2 | | 0.294 | | 91.4 |
| GelMA-3 | | 0.289 | | 89.8 |
| Mean ± SD | | 0.287 ± 0.008 | | 89.2 ± 2.5 |

**4. References**

[1] a) P. Fagerholm, N. S. Lagali, J. A. Ong, K. Merrett, W. B. Jackson, J. W. Polarek, E. J. Suuronen, Y. Liu, I. Brunette, M. Griffith, Biomaterials 2014, 35, 2420; b) K. Merrett, P. Fagerholm, C. R. McLaughlin, S. Dravida, N. Lagali, N. Shinozaki, M. A. Watsky, R. Munger, Y. Kato, F. Li, C. J. Marmo, M. Griffith, Invest Ophthalmol Vis Sci 2008, 49, 3887.

[2] A. Assmann, A. Vegh, M. Ghasemi-Rad, S. Bagherifard, G. Cheng, E. S. Sani, G. U. Ruiz-Esparza, I. Noshadi, A. D. Lassaletta, S. Gangadharan, A. Tamayol, A. Khademhosseini, N. Annabi, Biomaterials 2017, 140, 115.

[3] M. Zhu, Y. Wang, G. Ferracci, J. Zheng, N. J. Cho, B. H. Lee, Sci. Rep. 2019, 9, 6863.
